# pLDH level of clinically isolated *Plasmodium vivax* and detection limit of pLDH based malaria rapid diagnostic test

**DOI:** 10.1186/1475-2875-12-181

**Published:** 2013-06-03

**Authors:** Jin Woo Jang, Chi Hyun Cho, Eun Taek Han, Seong Soo A An, Chae Seung Lim

**Affiliations:** 1 Department of Laboratory Medicine, College of Medicine, Korea University Guro Hospital, Guro 2 Dong, Guro Gu, Seoul 152-703, Republic of Korea; 2 Department of Parasitology, Kangwon National University College of Medicine, Chuncheon, Republic of Korea; 3 College of Bionano Technology, Gachon Bionano Research Institute, Kyungwon University, Seongnam-si, Gyeonggi Do, Republic of Korea

**Keywords:** *Plasmodium vivax*, Standard deviation, OptiMAL, Sensitivity, Specificity, pLDH

## Abstract

**Background:**

The malaria rapid diagnostic tests (RDTs) are now widely used in the world. Compared to *Plasmodium falciparum*, a poor sensitivity of RDTs was reported against *Plasmodium vivax* based on the adopted antibody against pan-*Plasmodium* antigen lactate dehydrogenase (pLDH) or aldolase. Levels of pLDH were measured from patient with *P. vivax*, and the correlations between the levels of pLDH and the sensitivities of RDTs were analysed among Republic of Korea (ROK) isolates.

**Methods:**

Three RDTs, OptiMAL test, SD BIOLINE Malaria Ag P.f/Pan test, Humasis Malaria Pf/Pan antigen test, and the Genedia pLDH antigen ELISA were performed with blood samples from 152 febrile patients and 100 healthy controls.

**Results:**

Three malaria RDTs revealed sensitivities between 85.5 (131/152) and 86.8% (132/152) with highest sensitivity for the detection of *P.vivax* by pLDH antigen ELISA test (145/152, 95.4%) in comparison to traditional microscopy using Giemsa–stained slides. None of the healthy control tested positive by three RDTs or ELISA, indicating 100% specificity in their respective test. Levels of pLDH among Korean *P. vivax* isolates ranged between 0 ng/mL and 22,387.2 ng/mL (mean ± standard deviation 3,917.5 ± 6,120.9 ng/mL). The lower detection limits of three RDTs were between 25 and 50 ng/mL with artificially diluted samples. The moderate degree of correlation was observed between parasitaemia and concentrations of pLDH (r = 0.4, p < 0.05).

**Conclusion:**

The pLDH levels of *P. vivax* are the main explanation for the variations in the performance of pLDH-based RDTs. Therefore, comparing sensitivities of RDT may need to include targeted biomarker value of patients.

## Background

Malaria remains to be one of the most difficult infectious diseases to control. Even though many new efforts are being made to eradicate the disease, the incidences and prevalence of malaria does not decrease and is worsening in some incidents [1]. Early diagnosis and treatment are essential for reducing its morbidity and mortality and for this reason, rapid and accurate diagnosis of malaria is important. The Giemsa-stained thin and thick peripheral blood smears and PCR methods are acceptable as gold standard, but the relative long assay time and the requirement of staff training or special instruments are limitations that are not easy to overcome [2,3]. Hence, effective and rapid immunochromatographic tests with simple procedures were developed for diagnosing malaria [4,5]. Even though the immunochromatography-based kit has proven to be an easy and rapid method for the clinical diagnosis of malaria by inexperienced personnel, few antigens were considered as targets for adoption in malaria rapid diagnostic tests (RDTs) until now.

The major target antigens of malaria RDTs were specific histidine-rich protein 2 (PfHRP-2), and *Plasmodium* lactate dehydrogenase in *Plasmodium falciparum* and pan-specific *Plasmodium* lactate dehydrogenase and aldolase were used for detecting other human *Plasmodium* species. While sensitivities of currently available malaria assay kits in the market would fluctuate by field conditions, relatively poor sensitivities of RDTs for *Plasmodium vivax* with reactive antibodies to pan-*Plasmodium* antigen lactate dehydrogenase (pLDH) or aldolase was reported in comparison to RDTs for *P. falciparum*[2,6-8]. The sensitivity of malaria RDTs depended usually on parasitaemia of patients, however, a common discrepancy was observed in treatment monitoring cases with gametocytaemia [9]. Monitoring the treatment outcomes through pLDH-based tests could be limited [9].

Among malaria RDTs, pLDH-based malaria RDTs present better sensitivities, corresponding well to the respective levels of parasitaemia. The levels of pLDH reflect the presence of metabolically viable *P. vivax* parasites, since pLDH usually disappear rapidly within three to five days in the body [10]. Hence, pLDH-based malaria RDTs could also be used to monitor patient responses to anti-malarial therapy. Overall, pLDH is the preferred target in developing many malaria RDTs in the field. While the performances of malaria RDTs were evaluated according to parasitaemia levels of patients in many studies [10-14], it should be noted that the target of rapid kit is antigens, not parasite itself. Until now, distribution of pLDH levels in *P.vivax* infected patients and sensitivities of currently available malaria RDTs were not reported. In addition, the association from RDT results between pLDH levels and parasitaemia was not evaluated.

In the present study, levels of pLDH with pLDH specific ELISA test were measured in diagnosed patients with *P. vivax* in comparison with three simultaneous RDT tests. The results of both RDT and pLDH ELISA assays for *P. vivax* were analysed and compared against parasitaemia levels from Giemsa-stain microscopy for calculating the detection limit of pLDH-based RDT kits.

## Methods

### Subjects

A total of 252 samples, including 100 healthy controls, were collected between April 2009 and November 2012 at Korea University Hospital, Republic of Korea (ROK). Patients (n = 152) had fever or a recent history of fever within a week of their return from malaria-endemic regions. All patients and control subjects gave their informed written consent to participate in the study. This study was approved by the Ethical Committee of the Korea University Guro Hospital.

Thick and thin blood films were prepared for the diagnosis of malaria. The species and density of the plasmodial parasites were blindly determined by two experts in malaria diagnosis, using microscopic examination of Giemsa-stained thick and thin blood films. Parasitaemia was indirectly calculated by counting the parasite numbers per 200 white blood cells (WBC) in blood film, and the WBC counts from the automatic blood cell counter (Cell-Dyn 4000, Abbott Diagnostics, USA).

### Polymerase chain reaction (PCR)

Genomic DNA was extracted from frozen pellets using a blood genomic DNA extraction kit (Bio-Solution, ROK) and stored at −80°C. Circumsporozoite protein (CSP) genes of *P. vivax* (Belem strain, M11926) were amplified as previously described [8].

### Rapid diagnostic tests for malaria

The three RDTs, OptiMAL test, SD BIOLINE Malaria Ag Pf/Pan test and Humasis Malaria Pf/Pan antigen test, were used in this study. Humasis Malaria Pf/Pan antigen test kits (AMAL-7025) were provided by the manufacturer for the evaluation, and the OptiMAL-IT test (710024) and SD BIOLINE Malaria Ag Pf/Pan test (05FK60) kits were purchased from BioRad Inc (Hercules, USA) and SD Inc (Seoul, ROK), respectively. OptiMAL test incorporated pLDH from *P. falciparum* and pan-*Plasmodium*-specific pLDH from *P. vivax*, as major specific target antigens. The other two tests used HRP-II from *P. falciparum* and pan-*Plasmodium*-specific pLDH from P. vivax for the specific target antigens. The assays were performed following each manufacturer’s instructions. All RDTs were read by different technicians, who were blinded against the results from the other diagnostic techniques and RDTs. Detection limit of pLDH and parasitaemia in three RDTs from five individual kits was determined from artificially diluted *P. vivax* samples with known parasitaemia (8,700/μL) and pLDH level (5,000 ng/ml).

### pLDH level measurement by pLDH antigen ELISA test

Genedia malaria antigen ELISA test (Green Cross Co, ROK) was provided for the evaluation. The ELISA test was based on the quantification of pLDH in a whole blood sample with immobilized pLDH-specific capture antibody on 96-well plates. The ELISA test was performed, as recommended by the manufacturer. Briefly, 50 mL of EDTA whole blood and controls were added to each well with 100 mL of diluent. After one-hour incubation at 37°C, unbound materials were washed away with PBS-Tween 20, and 50 mL peroxidase-conjugated goat anti-pLDH (1:101 dilution) was added to each well. The supplied recombinant pLDH antigen on the kit was diluted serially from 2,500 to 0.02 ng/mL as positive controls and reference standards. After 30 min incubation at 37°C, the excess peroxidase-conjugated antibody was washed away three times with 500 mL PBS/0.05% Tween-20 (PBS/Tween), and the chromogenic signal was developed with addition of 200 mL tetramethylbenzidine-dimethyl sulphoxide (TMB). Following 20-min incubation at 37°C, the reaction was stopped by adding 20 μl of 5 N H2SO4. Absorbance at 450 nm was read using an ELISA reader (Behring Elisa Processor III, Siemens, Germany). Each sample was tested in duplicate, and the optical density (OD) values were averaged. The background value at wavelength at 650 nm was subtracted from the OD values at 450 nm for each sample.

The obtained OD values of the standard references were plotted against their concentrations, either on semi-logarithmic graph paper or using an automated method. The concentrations of the samples were read directly from the standard reference curve. Samples with higher concentrations than the highest standard reference were diluted, re-assayed, and calculated according to the dilution factor. For the data analysis of dichotomous groups, cut-off value (CO) for the positive ELISA signals was set at 0.1 plus mean OD of negative control (as recommended by manufacturer). S/CO value was counted as S divided by CO. If S/CO value was greater or less than 1.0 (≥1.0 or <1.0), the samples were interpreted as positive or negative, respectively.

## Results

**Figure 1:**
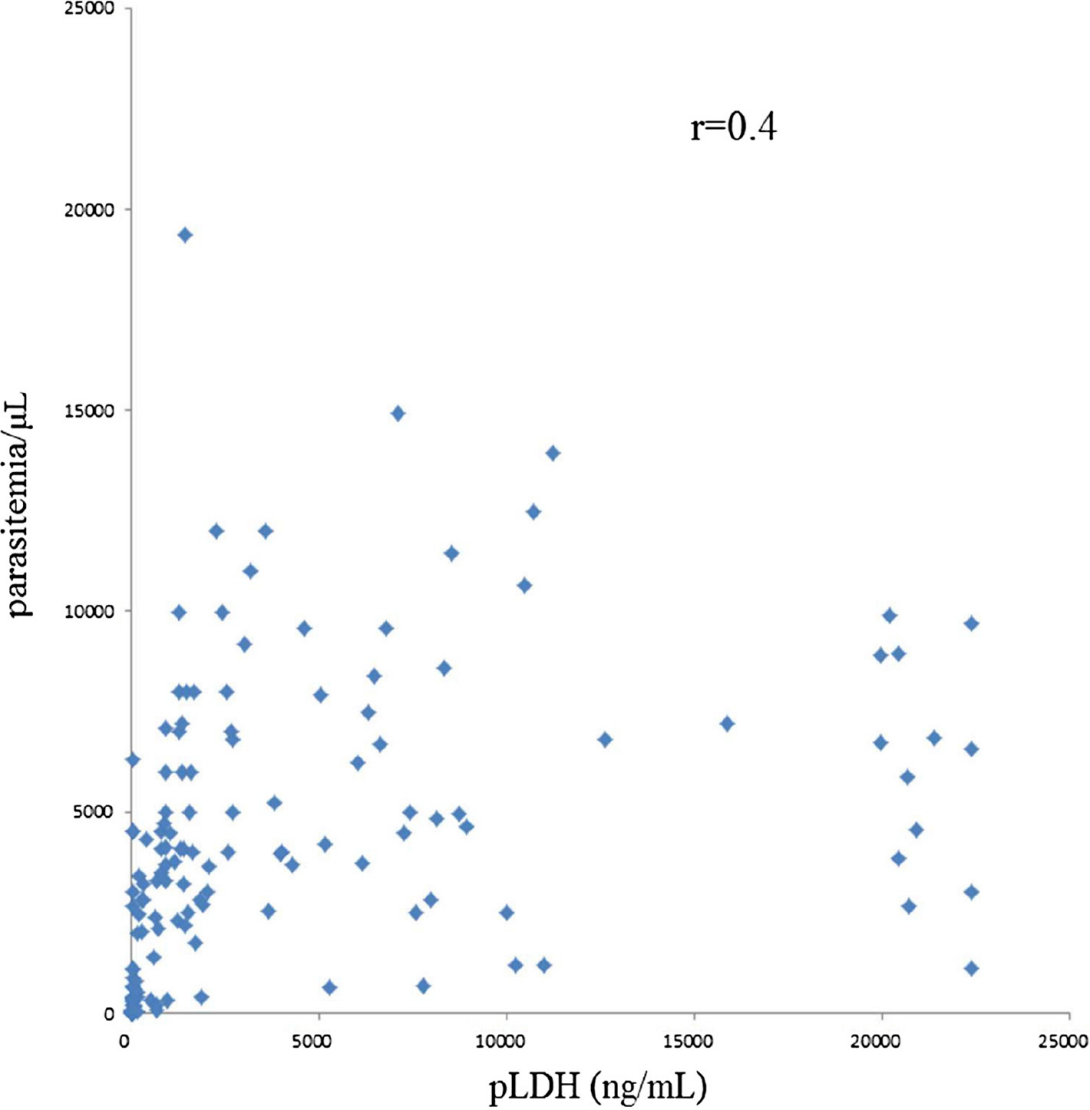
**Correlations between parasitaemia and the levels of pLDH among isolated *Plasmodium vivax *patients with clinical symptoms.**

During the study period, 152 patients and 100 healthy controls were enrolled and diagnosed for malaria parasites by PCR and microscopic examination of Giemsa-stained blood smear. CSP genes were successfully amplified from parasite genomic DNA from 152 patients. Mean (±SD) parasitaemia level was 3954.1 ± 3712.5/μL with range between 2.0 and 19,382.0/μL, and mean (±SD) pLDH level of *P. vivax* infected patients was 3,971.5 ± 6120.9 ng/mL with range between 0 and 22,387.2 ng/ml (Table 1). Major pLDH levels of *P. vivax* (38.8%, 59/152) fell between 500–5,000 ng/mL (mean ± SD, 1702.0 ± 1001.5 ng/mL), and the second frequent level was over 5,000 ng/mL (25.1%, 40/152). Thirty seven cases (20.1%) revealed extremely low pLDH levels, less than 50 ng/mL. In healthy controls, pLDH was not detected. The lowest detectable level of pLDH antigen ELISA was 6.3 ng/mL, and lowest parasitaemia level of patients detected by pLDH antigen ELISA was five parasites/μL in clinical samples. Direct correlation was observed between pLDH level and parasitaemia (r = 0.4, p < 0.05) (Figure 1). However, some discrepancies were observed between parasitaemia and pLDH levels (Figure 1). Seven cases of P. vivax patients with parasitaemia over 1,000/μL presented very low pLDH levels (<100 ng/mL).

**Table 1:** **The sensitivities of rapid diagnostic tests compared against the levels of pLDH in detecting *Plasmodium vivax *infection**

	**Sensitivity**				**Mean**	**Mean**
**pLDH (ng/mL)**	**pLDH Ag ELISA(%)**	**SD Bioline (%)**	**OptiMAL (%)**	**Humasis (%)**	**Parasitemia (/μL)**	**pLDH (ng/mL)**
>5,000 (n = 40)	40/40 (100.0)	40/40 (100.0)	40/40 (100.0)	40/40 (100.0)	6,265.1 ± 3,586.7	12,532.5 ± 6,286.6
500-5,000 (n = 59)	59/59 (100.0)	59/59 (100.0)	59/59 (100.0)	59/59 (100.0)	5,118.9 ± 3,463.8	1,702.0 ± 1,001.5
50-500 (n = 16)	16/16 (100.0)	14/16 (87.5)	14/16 (87.5)	14/16 (87.5)	1,613.4 ± 1,373.6	176.5 ± 105.7
<50 (n = 37)	30/37 (81.1)	19/37 (55.5)	18/37 (52.7)	19/37 (55.5)	722.1 ± 1344.6	13.5 ± 9.1
Total (n = 152)	145/152 (95.4)	132/152(86.8)	131/152 (85.5)	132/152 (86.8)	3,954.1 ± 3,712.5	3,971.5 ± 6,120.9
Healthy control	100	0/100	0/100	0/100	0	0

The diagnostic sensitivity of pLDH antigen ELISA was 95.4% (145/152) among microscopy-positive cases with 100% specificity (0/100), which was superior to three malaria RDTs. OptiMAL test, SD BIOLINE Malaria Ag P.f/Pan test, and Humasis Malaria Pf/Pan antigen test had sensitivity of 85.5% (131/152), 86.8% (132/152), and 86.8% (132/152) with each 100% specificity (0/100), respectively (Table 1).

From five repeated test by each kit, detection limits of pLDH in three RDTs were determined as 50 ng/mL for OptiMAL test and 25 ng/ml for SD BIOLINE Malaria Ag P.f/Pan test and Humasis Malaria P.f/Pan antigen test with artificially diluted samples (Table 2). The pLDH levels in negative cases by all three RDTs (n = 19) were approximately 19.7 ± 28.4 ng/mL, while values of parasitaemia were observed at 348.8 ± 602.4/μL. In all positive cases, much higher levels of pLDH from RDTs were observed at 4,651.2 ± 6374.1 ng/mL with corresponding parasitaemia of 4,586.1 ± 6,666.8/μL (Table 3).

**Table 2:** **Limit of detection for pLDH in three rapid diagnostic tests by using artificially diluted samples of *Plasmodium vivax***

**pLDH (ng/mL)**	**Parasitaemia(/μL)**	**SD Bioline (n = 5)**	**OptiMAL (n = 5)**	**Humasis(n = 5)**
5,000	8,700.0	100%	100%	100%
500	870.0	100%	100%	100%
250	435.0	100%	100%	100%
50	87.0	100%	100%	100%
25	44.0	100%	0%	100%
5	8.7	0%	0%	0%
0.5	0.9	0%	0%	0%
0	0	0%	0%	0%

**Table 3:** **The sensitivities of rapid diagnostic tests were compared to the levels of pLDH in detecting *Plasmodium vivax *infection**

		**Mean**	**Mean**
**RDT**	**N**	**Parasitaemia(/μL)**	**pLDH (ng/mL)**
All RDT Positive	130	4,586.1 ± 6,666.8	4,651.2 ± 6,374.1
2 RDT positive	3	777.7 ± 1076.5	5.0 ± 7.1
All RDT Negative	19	348.8 ± 602.4	19.7 ± 28.4
Total	152	3,954.1 ± 3,712.5	3,971.5 ± 6,120.9

## Discussion

Malaria diagnostic tests should be easy to perform and the interpretation of the results by examiners needs to be simple, however, in many RDTs sensitivities and specificities fluctuate in various field settings. The evaluations of pLDH-based RDTs for *P. vivax* indicate that quality and performance are not satisfactory due to low sensitivity. Poor manufacturing conditions or inadequate precautions in transportation and storage of RDTs result in low sensitivity and reproducibility [11,12].

Even though patient parasitaemia levels are the most important determining factor for sensitivity of RDTs, several exceptional cases could not be explained by parasitaemia factors only. Traditionally, the performances of malaria RDTs are analysed by comparing the value with the parasitaemia levels from microscopic examinations. Microscopy-based evaluations reveal frequent limitations due to incorrect interpretation results dependent on examiners’ training and experience. Hence, the measured value of pLDH by ELISA could be more objective than the parasitaemia value, and the value of pLDH could provide more reliable analysis of selecting adequate malaria RDTs.

Without adequate baseline data of pLDH levels from clinically isolated *P. vivax*, it is difficult to correct the interpretation of results to make better decisions for selecting reliable malaria RDTs. Since none of the malaria RDTs detect the parasite itself, the secreted biomarkers became the diagnostic targets. To date, the level of target biomarkers using a standardized protocol for clinically isolated malaria samples are rarely reported [13].

Here, In this study, the distribution of pLDH in clinically isolated *P. vivax* samples, and the relationships between levels of pLDH and parasitaemia and between levels of pLDH and sensitivities of pLDH-based RDTs are assessed. In WHO/FIND malaria RDT testing round 4 report, Humasis Malaria P.F/Pan Antigen test (AMAL-7025) showed panel detection score (PDS) of 0% at both 200 and 2000 parasites/μL of *P. vivax*[14]. In addition, false positive rate of Humasis kit was 97.8%. While the performance parameter in this study was not PDS, but clinical sensitivity, Humasis kit in this study demonstrated the clinical sensitivity of 55.5 ~ 100.0% at similar parasitaemia (Table 1). Next, specificity of Humasis kit for *P. vivax* was 100% with no observed false positivity. As for OptiMAL-IT kit (710024), round 4 report presented PDS of 97.1 and 68.6% at 200 and 2,000 ~ 5,000 parasites/μL of *P. vivax*, respectively. On the other hand, OptiMAL-IT showed increased clinical sensitivity from 52.7 to 100%, as the *P. vivax* parasitaemia increased. As for the SD Bioline Malaria Ag P.f/Pan kit (05 F60), round 4 report showed PDS of 97.1 and 100.0% at 200 and 2,000 ~ 5,000 parasites/μL of *P. vivax*, respectively. However, SD Bioline demonstrated slightly decreased clinical sensitivity of 87.5 ~ 100.0% (Table 1). In this study, lower sensitivities in all three RDTs were observed from groups with lower pLDH antigen levels (Table 1). From the study for determining the limit of detection, lower levels pLDH associated with lower sensitivities in all three RDTs (Table 2). These direct correlations were similar to the levels of parasitaemia in all three RDTs. Above results suggested the possibility of pLDH, as a performance parameter for malaria RDTs. This information on currently available RDTs is valuable in revealing detection limits against pLDH. Therefore, the current results could provide one more reasonable protocol for comparing various malaria RDTs and their practical guidelines in developing sensitive malaria RDTs.

pLDH antigen ELISA system showed higher sensitivity (95.4%) in comparison with three RDTs (85.5-86.8%), which might be associated with the fact that ELISA uses 50 μL of blood, while RDTs used only 5–10 μL of blood. RDTs are fast and convenient diagnostic tools. Parasitaemia levels of *P. vivax* in ROK ranged 2–25,000/μL with mean level of 3,954.1 ± 3712.5/μL, while pLDH levels of *P. vivax* ranged between 0–22,387 ng/mL with mean value of 3,971.5 ± 6120.9 ng/mL. pLDH levels showed moderate correlation with parasite density of P. vivax, as determined by microscopy (r = 0.4, p < 0.05).

From three tested RDTs, pLDH levels and parasitaemia levels of three RDTs, positive cases were higher than two RDT positive cases or all RDT negative cases. One positive sample from two RDT tests (n = 3) showed disparity between parasitaemia and pLDH levels, where the pLDH and parasitaemia levels were 15 ng/mL and 2,300/μL, respectively. These levels of parasitaemia most likely produce clinical symptoms. RDTs are fast and convenient diagnostic tools. Using only RDTs, malaria diagnosis might be difficult and patients might be left untreated, leading to severe complications. Since the evaluation of RDTs is currently based on patients’ parasitaemia levels, with little information on targeted biomarkers in clinical isolates, the current study provides additional information for selecting adequate malaria RDT in fields.

## Competing interests

The authors have declared that they have no competing interests.

## Authors’ contributions

JWJ carried out the molecular work and RDT test. CHC carried out the immunoassays. ETH & SSA participated in the design of the study and performed the statistical analysis. CSL contributed to the design and coordination of the study, assisted with data entry, analysis and interpretation. All authors read and approved the final manuscript.
